# NG2 glial cells regulate neuroimmunological responses to maintain neuronal function and survival

**DOI:** 10.1038/srep42041

**Published:** 2017-02-14

**Authors:** Masayuki Nakano, Yasuhisa Tamura, Masanori Yamato, Satoshi Kume, Asami Eguchi, Kumi Takata, Yasuyoshi Watanabe, Yosky Kataoka

**Affiliations:** 1 Cellular Function Imaging Team, Center for Life Science Technologies, RIKEN, 6-7-3 Minatojima-minamimachi, Chuo-ku, Kobe 650-0047, Japan; 2 Department of Physiology, Osaka City University Graduate School of Medicine, 1-4-3 Asahimachi, Abeno-ku, Osaka 545-8585, Japan; 3 Multi-Modal Microstructure Analysis Unit, RIKEN CLST-JEOL Collaboration Center, RIKEN, 6-7-3 Minatojima-minamimachi, Chuo-ku, Kobe 650-0047, Japan; 4 Pathophysiological and Health Science Team, Center for Life Science Technologies, RIKEN, 6-7-3 Minatojima-minamimachi, Chuo-ku, Kobe 650-0047, Japan

## Abstract

NG2-expressing neural progenitor cells (i.e., NG2 glial cells) maintain their proliferative and migratory activities even in the adult mammalian central nervous system (CNS) and produce myelinating oligodendrocytes and astrocytes. Although NG2 glial cells have been observed in close proximity to neuronal cell bodies in order to receive synaptic inputs, substantive non-proliferative roles of NG2 glial cells in the adult CNS remain unclear. In the present study, we generated NG2-HSVtk transgenic rats and selectively ablated NG2 glial cells in the adult CNS. Ablation of NG2 glial cells produced defects in hippocampal neurons due to excessive neuroinflammation via activation of the interleukin-1 beta (IL-1β) pro-inflammatory pathway, resulting in hippocampal atrophy. Furthermore, we revealed that the loss of NG2 glial cell-derived hepatocyte growth factor (HGF) exacerbated these abnormalities. Our findings suggest that NG2 glial cells maintain neuronal function and survival via the control of neuroimmunological function.

Tissue-specific stem/progenitor cell differentiation maintains various organ tissues. In the central nervous system (CNS), neural progenitor cells expressing chondroitin sulfate proteoglycan 4 (NG2), which are known as NG2 glial cells (or oligodendrocyte progenitor cells), represent 5–8% of all cells in the adult CNS^1^. Such cells are organized in a grid-like or tiled manner, with individual cells occupying non-overlapping domains^2^. NG2 glial cells migrate from the germinal zones, actively proliferate, and differentiate into oligodendrocytes to form myelinated tracts during early postnatal life^3^. The cells continue to give rise to oligodendrocytes under normal physiological conditions^4^, even in adulthood.

NG2 glial cells comprise the majority of the proliferative cells in the adult CNS^1^ and can rapidly balance proliferation and migration to restore their density in response to focal cellular loss^4^, particularly in such conditions as acute CNS injury^5^ and chronic neurodegenerative disease^3^,^6^. In the cerebral cortex and hippocampus, NG2 glial cells are frequently found in close proximity to dendrites and neuronal cell bodies^7^,^8^,^9^. Moreover, these cells receive direct synaptic input from glutamatergic^10^ and GABAergic^11^ neurons. Sustained activation of AMPA^12^ and GABA^13^ receptors has been observed to regulate the proliferation and migration of NG2 glial cells. Such observations imply that NG2 glial cells have an important role in the adult CNS beyond that of cellular reproduction. Sakry *et al*.^14^ reported that NG2 glial cells may modulate the neuronal network via bidirectional cross-talk with surrounding neurons. Moreover, the proliferative activity and migration ability of NG2 glial cells gradually decline with age^15^,^16^,^17^. In NG2 glial cells, the upregulation of esophageal cancer-related gene 4 (Ecrg4) during cellular aging induced a decline of proliferative activity^18^. In addition, abnormal proliferative and differentiating activity of NG2 glial cells is involved in a number of age-related neurodegenerative diseases^19^ and demyelinating diseases^20^. Such findings support the hypothesis that NG2 glial cells maintain the neural environment under normal physiological conditions, and that the dysfunction of these cells leads to an impairment of neuronal function and neurodegeneration.

To test this hypothesis, we generated transgenic rats expressing herpes simplex virus thymidine kinase (HSVtk) under the control of the promoter for NG2 (NG2-HSVtk Tg rats). HSVtk is a suicide gene that converts antiviral nucleoside analog prodrugs such as ganciclovir (GCV) into a toxic triphosphate molecule that can be incorporated into the genome and subsequently terminate DNA synthesis. Therefore, this manipulation may allow for selective ablation of proliferative NG2 glial cells. The HSVtk/GCV system has been used to reveal substantive roles for various cell types in the CNS, including astrocytes^21^, microglia^22^, and neuronal stem cells^23^,^24^. Thus, the present study aimed to use the HSVtk/GCV ablation system to reveal substantive roles for NG2 glial cells in adult mammalian neuronal function. Our results show that ablation of NG2 glial cells impaired neuronal function and induced neuronal cell death due to excessive neuroinflammation. Furthermore, our findings suggest that NG2 glial cells suppress neuroinflammation and support the survival of hippocampal neurons through the production of growth factors including hepatocyte growth factor (HGF).

## Results

### HSVtk is selectively expressed in NG2-HSVtk transgenic rats

**Figure 1:**
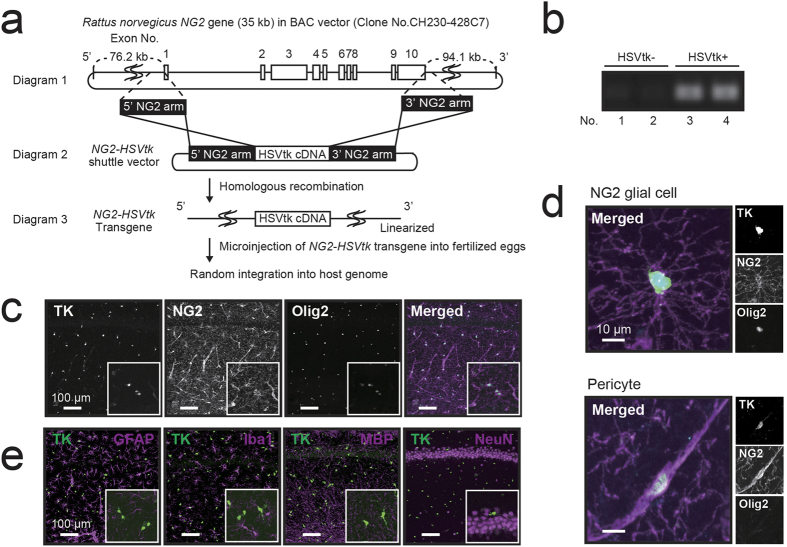
Characterization of NG2-HSVtk transgene and transgenic rats. (**a**) Schematic diagram of the engineered NG2-HSVtk BAC construct. Diagram 1 depicts the structure of the Rattus norvegicus NG2 gene (not to scale). Exons are indicated as white blocks. The regions used for 5′ and 3′ homology arms are indicated. Diagram 2 depicts the structure of the NG2_HSV-TK shuttle vector that was used to modify the BAC DNA in order to insert the HSV-TK cDNA into the BAC clone that contained the NG2 gene in the center. Diagram 3 depicts the structure of the modified BAC DNA that was used for microinjection into fertilized oocytes. (**b**) PCR genotyping of tail DNA from NG2-HSVtk transgenic rats. (**c**) Confocal images (magnified views in white boxes) of immunoreactivity for HSVtk (TK, green in the merged image), NG2 (NG2, magenta in the merged image) and Olig2 (Olig2, cyan in the merged image) in the hippocampus of NG2-HSVtk transgenic rats. (**d**) Confocal image showing a polydendritic NG2 glial cell that was immunopositive for HSVtk, NG2, and Olig2; and also showing a pericyte that was immunopositive for HSVtk and NG2 but not for Olig2. (**e**) Confocal images (magnified views in white boxes) of glial fibrillary acidic protein (GFAP, a marker of astrocytes), Iba1 (a marker of microglia), myelin basic protein (MBP, a marker of oligodendrocyte), and NeuN (a marker of neuronal nuclei), with TK (HSVtk) in the hippocampus of NG2 transgenic rats. Scale bars represent 100 μm (**c**,**e**) and 10 μm (**d**).

To uncover the non-proliferative functions of NG2 glial cells, we generated bacterial artificial chromosome (BAC) transgenic rats expressing HSVtk under the control of the NG2 promoter (Fig. 1a). Transgenic rats were identified using polymerase chain reaction (PCR) genotyping of tail DNA (Fig. 1b). The expression of HSVtk was ascertained via immunohistochemical staining (Fig. 1c). Almost all NG2-positive cells expressed HSVtk in the adult brain (Fig. 1c). NG2 and HSVtk expressing cells were widely distributed in the hippocampus (Fig. 1c), parietal cortex, corpus callosum, striatum, thalamus, hypothalamus, and amygdala (Supplementary Fig. S1). NG2 was expressed not only in glial cells but also in vascular mural cells known as pericytes. NG2 glial cells are defined as polydendritic cells that express NG2 and Olig2 (Fig. 1d). In contrast, pericytes are NG2+ and Olig2- bipolar cells that are primarily localized in blood vessels (Fig. 1d). To evaluate the expression rate of NG2 glial cells in HSVtk positive cells, we quantified the number of NG2 glial cells (Olig2+/HSVtk+ [81.9%; 2,424 cells]) and pericytes (Olig2-/HSVtk+ [18.1%; 536 cells]) in the same area (per mm^2^). No significant difference was observed in the distribution of NG2 glial cells among hippocampal regions (Supplementary Fig. S2b, Control). Further, we did not observe colocalization of HSVtk with other cell-specific markers for astrocytes (glial fibrillary acidic protein, GFAP), microglia (Iba1), oligodendrocytes (myelin basic protein, MBP), and neurons (NeuN) (Fig. 1e). Such observations indicate that HSVtk was selectively expressed in both NG2 glial cells and pericytes in NG2-HSVtk transgenic rats.

### GCV selectively ablates NG2 glial cells in NG2-HSVtk transgenic rats

**Figure 2:**
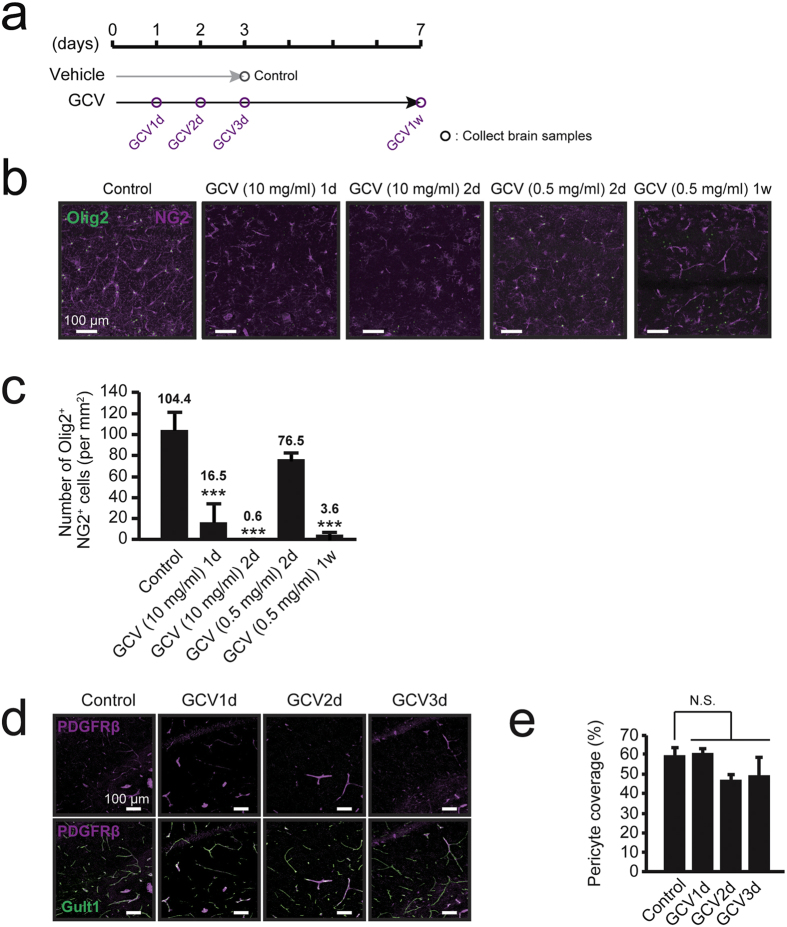
Ablation of NG2 glial cells in the hippocampus of NG2-HSVtk transgenic rats. (**a**) Timeline of the experimental design for the treatment with vehicle and ganciclovir (GCV). (**b**) Confocal images of immunoreactivity for Olig2 (Olig2, green) and NG2 (NG2, magenta) in NG2-HSVtk transgenic rats treated with vehicle (Control) or GCV at doses of 10 and 0.5 mg/ml for 1, 2 or 7 days [GCV (10 mg/ml) 1d, GCV (10 mg/ml) 2d, GCV (0.5 mg/ml) 2d, GCV (0.5 mg/ml) 1w]. (**c**) The number of NG2 glial cells (immunopositive cells for Olig2 and NG2) in animals treated with vehicle or GCV. Mean ± SD, n = 3 rats [Control, GCV (0.5 mg/ml) 2d, GCV (0.5 mg/ml) 1w] or 5 rats [GCV (10 mg/ml) 1d, GCV (10 mg/ml) 2d]; ***p < 0.001, based on a one-way analysis of variance (ANOVA) followed by Tukey-Kramer test. (**d**) Confocal images of immunoreactivity for PDGFRβ (a marker of pericytes; magenta) and Glut1 (a marker of endothelial cells; green) in animals treated with vehicle or GCV for 1, 2, or 3 days. (**e**) Percentages of pericyte coverage in animals treated with vehicle or GCV. The pericyte coverage was determined as a ratio (%) of PDGFRβ-positive area on the Glut1-positive capillary to the total Glut1-positive area. Mean ± SD, n = 3 rats (Control, GCV1d, GCV2d, and GCV3d); N.S., non-significant, p > 0.05, based on a one-way ANOVA followed by Tukey-Kramer test (**c**,**e**). Scale bars represent 100 μm (**b**,**d**).

We selected the dose of GCV in reference to previous cell ablation studies using transgenic mice^22^,^23^,^24^. We calculated the GCV dose for rats knowing that the volume of the rat brain is on average 4.5 times larger than that of the mouse brain^25^. We performed intracerebroventricular infusion of GCV at a dose of 10 mg/ml into the left lateral ventricle of wild-type (WT) rats and NG2-HSVtk transgenic rats. Immunohistochemical staining revealed that GCV infusion did not change the number of NG2 glial cells, pericytes, microglia, astrocytes, and neurons in WT rats (Supplementary Fig. S3). In contrast, NG2 glial cells were ablated in the surrounding regions of the lateral ventricle, including the parietal cortex, corpus callosum, striatum, hippocampus, and thalamus 1 day after the start of GCV infusion in NG2-HSVtk transgenic rats. Notably, the ablation rate of NG2 glial cells was higher on the ipsilateral side than the contralateral side. Morphological changes suggesting neuronal impairment were also observed in the region showing NG2 glial cell ablation, the most dramatic changes with regard to neuronal atrophy were observed in the hippocampus 4 days after the start of GCV infusion, particularly on the ipsilateral side (Supplementary Fig. S2a). Histological analysis revealed a complete ablation of NG2 glial cells throughout the hippocampal region (Supplementary Fig. S2b) within 2 days after the start of GCV infusion, 16.5 ± 17.1 cells/mm^2^ [vs. GCV (10 mg/ml) 1d; p < 0.001] and 0.6 ± 0.5 cells/mm^2^ [vs. GCV (10 mg/ml) 2d; p < 0.001] compared with 104.4 ± 15.8 cells/mm^2^ in the hippocampi of control animals (Fig. 2b and c). A large amount of NG2 cell debris was observed in dying NG2 cells in the hippocampus on days 2 and 3 after the start of GCV infusion (Fig. 2b). No significant difference was observed in the ablation rate of NG2 glial cells among hippocampal regions (Supplementary Fig. S2b). In addition, we administered lower amounts of GCV in NG2-HSVtk transgenic rats. Histological analysis revealed that the number of NG2 glial cells was gradually reduced in the hippocampus 2 days after the start of GCV infusion in dose-dependent manner (Supplementary Fig. S2c). One week after the start of GCV infusion at a dose of 0.5 mg/ml, NG2 glial cells were ablated to the same extent as that observed after treatment with 10 mg/ml of GCV for 2 days compared with control (Fig. 2b and c). NG2 glial cells complete the differentiation into mature oligodendrocytes taking a few weeks^2^,^26^. To eliminate the influences of dysfunctional differentiation, we used a conventional dose of GCV (10 mg/ml) to ablate them in the shorter period.

As aforementioned, HSVtk was expressed not only in NG2 glial cells but also in pericytes. In mature blood vessels in the brain, pericyte proliferative is low. Therefore, the pericytes expressing HSVtk in the present study likely had little sensitivity to GCV. We confirmed this idea by quantifying pericytes through the identification of platelet-derived growth factor receptor beta (PDGFRβ)-immunopositive cells in the population of endothelial cells expressing Glut1 (pericyte coverage) in the hippocampus^27^. Predictably, there was no significant difference in the rate of pericyte coverage in the hippocampus between the control and GCV treatment groups (Fig. 2d and e; p = 0.065). Such observations indicate that NG2 glial cells were selectively ablated after GCV infusion in the transgenic rats.

### Ablation of NG2 glial cells induces hippocampal cell death

**Figure 3:**
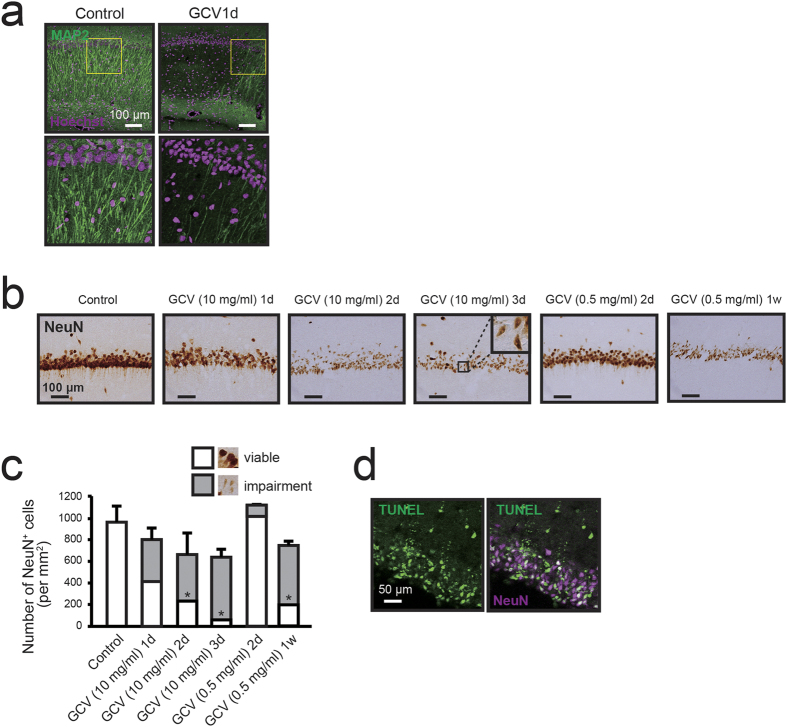
Ablation of NG2 glial cells induces neurodegeneration in the hippocampus. (**a**) Confocal images of immunoreactivity for microtubule-associated protein 2 (MAP2, green) in NG2-HSVtk transgenic rats treated with vehicle (Control) or GCV at a dose of 10 mg/ml for 1 day (GCV1d). Lower panels show magnified views of those images presented in yellow boxes in the upper panels. Hoechst, cell nuclear staining. (**b**) Bright-field immunohistochemical observations of NeuN in the hippocampal CA1 region of NG2-HSVtk transgenic rats treated with vehicle or GCV at doses of 10 and 0.5 mg/ml for 1, 2, 3 or 7 days [GCV (10 mg/ml) 1d, GCV (10 mg/ml) 2d, GCV (10 mg/ml) 3d, GCV (0.5 mg/ml) 2d, GCV (0.5 mg/ml) 1w]. Black boxes in GCV (10 mg/ml) 3d show magnification of the microscopic view. (**c**) The number of NeuN positive cells in the hippocampus of NG2-HSVtk transgenic rats treated with vehicle or GCV at doses of 10 and 0.5 mg/ml for 1, 2, 3 or 7 days. Photomicrographs indicate representative images for viable (viable, white) and damaged neurons (impairment, grey). (**d**) Confocal images showing TUNEL (green) and immunoreactivity for NeuN (magenta) in the hippocampal CA2 region of NG2-HSVtk transgenic rats treated with GCV at a dose of 10 mg/ml for 3 days. Mean ± SD, n = 3 rats [Control, GCV (10 mg/ml) 3d, GCV (0.5 mg/ml) 2d, GCV (0.5 mg/ml) 1w] or 5 rats [GCV (10 mg/ml) 1d, GCV (10 mg/ml) 2d]; *p < 0.05, based on a one-way ANOVA followed by Tukey-Kramer test. Scale bars represent 100 μm (**a**,**b**) and 50 μm (**d**).

To study the influence of NG2 glial cell ablation on neurons, we examined morphological changes based on the immunostaining of microtubule-associated protein 2 (MAP2) and NeuN in the hippocampus. Immunohistochemical analysis revealed that neuronal dendrites began to exhibit impairments just 1 day after the start of GCV infusion at a dose of 10 mg/ml (Fig. 3a). Neuronal nuclei were also condensed and disrupted in the hippocampus (Fig. 3b). The total number of intact and impaired NeuN-positive cells was reduced by 33.7% on day 3 after GCV infusion at a dose of 10 mg/ml (Fig. 3c). Among these NeuN-positive cells, reductions in the number of viable hippocampal neurons were observable beginning 1 day after the start of GCV infusion at a dose of 10 mg/ml (417.9 ± 392.8 cells/mm^2^; p = 0.09), with significant decreases evident 2 days after the initial observation (237.7 ± 280.9 cells/mm^2^; p = 0.021), in comparison to the number observed in control animals (963.4 ± 147.5 cells/mm^2^) (Fig. 3c). In the study using lower doses (~1.0 mg/ml) of GCV in NG2-HSVtk transgenic rats, we hardly observed the degeneration of hippocampal neurons even at 2 days after the start of GCV infusion (Fig. 3b,c). One week after the start of GCV infusion at a dose of 0.5 mg/ml, the total number of intact and impaired NeuN-positive cells was reduced to the same extent as that observed after treatment with 10 mg/ml of GCV for 2 days (Fig. 3c). Histological analysis further revealed that the degeneration of neurons occurred throughout the hippocampus (Supplementary Fig. S4a,b). Such neuronal loss and degeneration occurred via the induction of apoptosis, as determined by a TdT-mediated dUTP nick end labeling (TUNEL) assay (Fig. 3d). Some TUNEL-positive cells did not appear to express NeuN, which may have been due to a gradually decrease in NeuN expression in degenerating neurons. In addition, the location and nuclear size of the TUNEL positive cells indicated that these cells appeared to be mainly neurons. Such results suggest that the ablation of NG2 glial cells induced apoptotic cell death in hippocampal neurons.

### Excessive neuroinflammation induces hippocampal neuronal apoptosis

**Figure 4:**
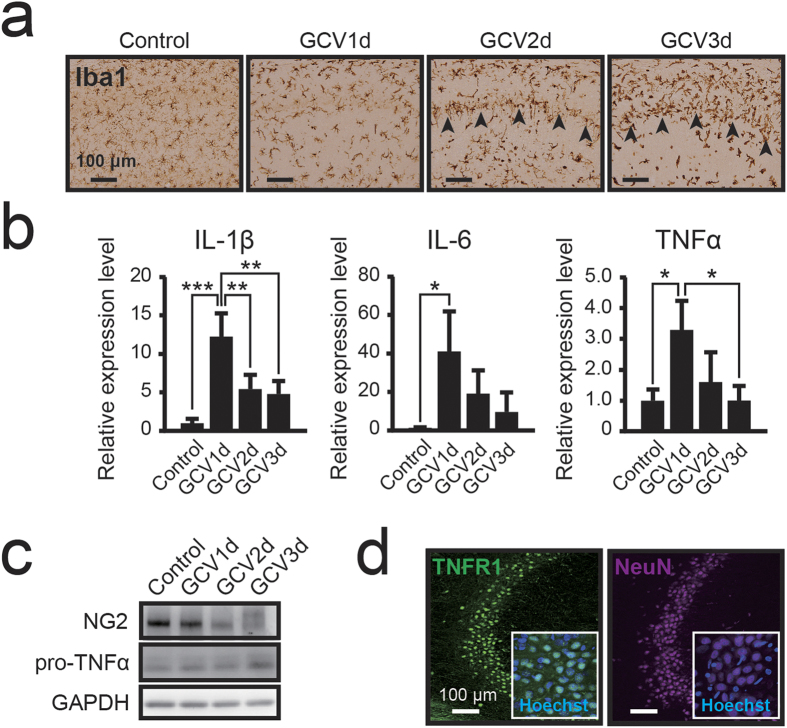
Ablation of NG2 glial cells induces neuroinflammation and activates microglia in the hippocampus. (**a**) Bright-field immunohistochemical observations of Iba1 in NG2-HSVtk transgenic rats treated with vehicle (Control) or GCV for 1, 2, or 3 days (GCV1d, GCV2d, GCV3d). Black arrowheads indicate the CA1 pyramidal cell layer. (**b**) Relative expression levels of interleukin-1β (IL-1β), interleukin-6 (IL-6), and tumor necrosis factor α (TNFα) mRNAs in the hippocampal tissue of NG2-HSVtk transgenic rats treated with vehicle or GCV for 1, 2, or 3 days. (**c**) Immunoblotting of NG2 and precursor protein of TNFα (pro-TNFα) in the hippocampal tissue of NG2-HSVtk transgenic rats treated with vehicle or GCV for 1, 2, or 3 days. Glyceraldehyde 3-phosphate dehydrogenase (GAPDH) was used as a loading control. (**d**) Confocal images (magnified views in white boxes) of immunoreactivity for TNF receptor 1 (TNFR1) and NeuN in the hippocampal CA2 region in NG2-HSVtk transgenic rats. Mean ± SD, n = 3 rats (Control, GCV3d) and 4 rats (GCV1d, GCV2d); *p < 0.05, **p < 0.01 or ***p < 0.001, based on a one-way ANOVA followed by Tukey-Kramer test. Scale bars represent 100 μm (**a,d**).

Hippocampal neurons are sensitive to various stressors, such as ischemia, hypoxia, and neuroinflammation^28^, and CA1 pyramidal neurons in particular are most vulnerable to ischemia, selectively undergoing apoptotic cell death^29^. Additionally, chronic neuroinflammation associated with activated microglia has been frequently reported to cause neurodegeneration and neuronal cell death in all regions of the hippocampus^30^. Similarly, neuronal cell death was observed throughout the hippocampus in the present study (Supplementary Fig. S4a,b). We assessed neuroinflammation following the widespread ablation of hippocampal NG2 glial cells. To examine microglial activation, we performed immunostaining for Iba1 in the NG2 glia-ablated hippocampus. While the retraction of microglial processes indicating activation-related morphological changes was observed 1 day after the start of GCV infusion, many microglia exhibited a rod-type or amoeboid morphology after an additional 2 or 3 days (Fig. 4a). Significant numbers of these rod-type microglia engulfed apoptotic neurons in the pyramidal cell layer of the hippocampus (arrowheads in Fig. 4a). During chronic activation of microglia, sustained exposure of neurons to pro-inflammatory cytokines induces neuronal dysfunction and cell death^30^. Therefore, we also examined pro-inflammatory gene expression in the hippocampus using real time PCR analysis. The expressions of IL-1β (p < 0.001), IL-6 (p = 0.019), and tumor necrosis factor alpha (TNFα; p = 0.016) were significantly upregulated 1 day after the start of GCV infusion, and gradually returned to baseline thereafter (Fig. 4b). IL-1β activation of the IL-1 receptor type 1 (IL-1R1) leads to NF-κB activation, which, in turn, increases the expression of IL-6 and TNFα^31^. Research suggests that TNFα activates the TNF receptor type 1 (TNFR1) to induce caspase-dependent apoptosis in acute phases of CNS insults^32^. In the present study, western blot analysis indicated that the expression of TNFα precursors was increased depending on the level of NG2 reduction (Fig. 4c). Moreover, immunohistochemical observation revealed that TNFR1 was expressed in hippocampal neurons (Fig. 4d). Such results suggest that ablation of NG2 glial cells activated microglia and induced excessive neuroinflammation.

**Figure 5:**
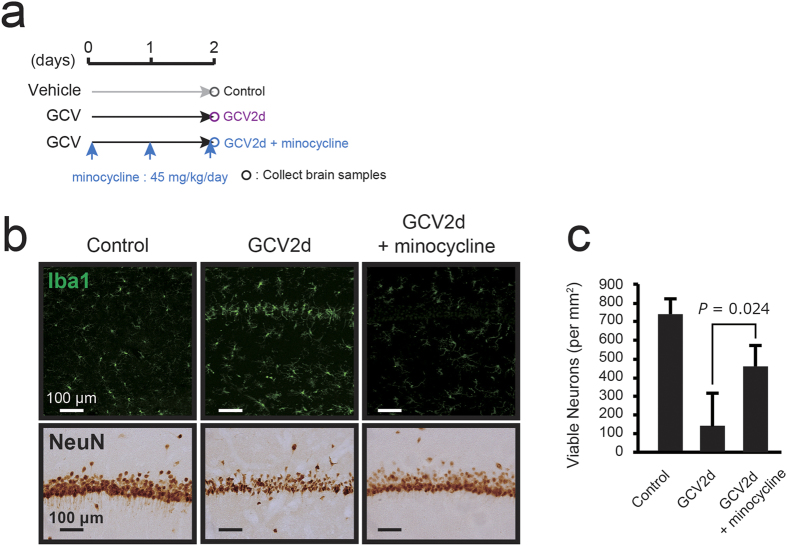
Inhibition of microglial activation attenuates the NG2 glial cell ablation-induced hippocampal cell death. (**a**) Timeline of the experimental design for the treatment with minocycline. (**b**) Immunohistochemical observations of Iba1 (upper panels) and NeuN (lower panels) in the hippocampal CA1 region of NG2-HSVtk transgenic rats treated with vehicle (Control), GCV for 2 days (GCV2d), or co-treatment with GCV and minocycline (45 mg/kg/day) for 2 days (GCV2d + minocycline). (**c**) The number of viable CA1 pyramidal neurons in NG2-HSVtk transgenic rats treated with vehicle (n = 3), GCV for 2 days (n = 6), or co-administration of GCV and minocycline (n = 3). p = 0.024, based on a the Student’s t-test analysis. Scale bars represent 100 μm (**b**).

**Figure 6:**
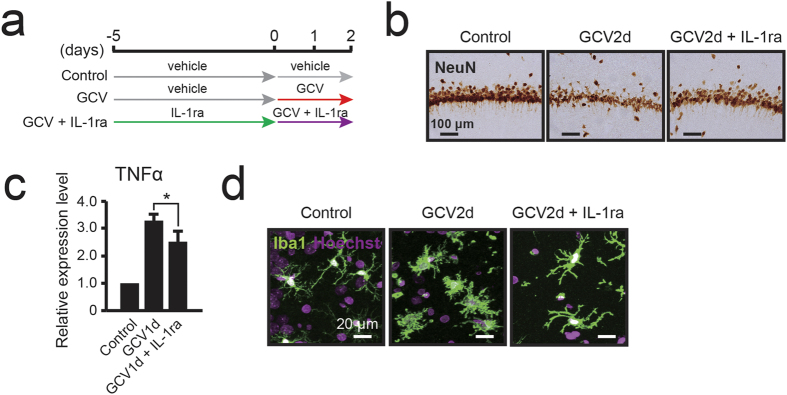
Suppression of IL-1β pro-inflammatory pathway attenuates the NG2 glial cell ablation-induced hippocampal cell death. (**a**) Timeline of the experimental design for the treatment with rat recombinant IL-1 receptor antagonist (rrIL-1ra). (**b**) Bright-field immunohistochemical observations of NeuN in the hippocampal CA1 region of NG2-HSVtk transgenic rats treated with vehicle (Control), GCV for 2 days (GCV2d), or co-administration of GCV and IL-1ra (1 μg/day) for 2 days following IL-1ra administration for 5 days (GCV2d + IL-1ra). (**c**) Relative expression levels of TNFα mRNAs in the hippocampi of NG2-HSVtk transgenic rats treated with vehicle (Control, value set as 1.0), GCV for 1 day, or co-administration of GCV and IL-1ra for 1 day following IL-1ra administration for 5 days. (**d**) Confocal images depicting microglia (Iba1, green) in the hippocampus CA1 region of NG2-HSVtk transgenic rats treated with vehicle, GCV for 2 days, or co-administration of GCV and IL-1ra for 2 days following IL-1ra administration for 5 days. Hoechst, cell nuclear staining. Mean ± SD, n = 2 rats (Control) and 3 rats (GCV1d, GCV2d, GCV1d + IL-1ra, GCV2d + IL-1ra); *p < 0.05, based on a Student’s t-test analysis. Scale bars represent 100 μm (**b**) and 20 μm (**d**).

To investigate the mechanism of neuronal degeneration, we suppressed the activation of microglia via co-treatment with minocycline (Fig. 5a), which is commonly used to inhibit microglial activation^33^. Histological analysis revealed that minocycline treatment inhibited the activation of microglia based on their morphological changes (Fig. 5b). Many hippocampal neurons were rescued 2 days after the start of GCV under co-treatment with minocycline (viable neurons, 462.1 ± 109.4 cells/mm^2^; p = 0.024) compared with those observed after infusion of GCV alone (viable neurons, 142.0 ± 174.9 cells/mm^2^) (Fig. 5b,c). These results suggest that neuronal degeneration was the result of microglia activation in the NG2 glia-ablated hippocampus. Next, we assessed in greater detail whether the neuroinflammation induced by the ablation of NG2 glial cells leads to apoptotic neuronal cell death, we attempted to inhibit the IL-1β pro-inflammatory pathway via co-administration of rat recombinant IL-1 receptor antagonist (rrIL-1ra), an endogenous competitive antagonist for the IL-1 receptor^34^ (Fig. 6a). We examined the effect of IL-1ra on the neuronal degeneration induced by the ablation of NG2 glial cells. Immunohistochemical staining for NeuN revealed that many hippocampal neurons were preserved 2 days after the start of GCV under co-treatment with IL-1ra (viable neurons, 755.9 ± 100.6 cells/mm^2^; p = 0.007) compared with those observed after infusion of GCV alone (viable neurons, 207.9 ± 165.9 cells/mm^2^) (Fig. 6b). We confirmed the effect of IL-1ra on TNFα expression using real time PCR analysis in the hippocampus. Administration of rrIL-1ra significantly suppressed the expression of TNFα (Fig. 6c; p = 0.032). Immunostaining for Iba1 revealed that the activation of microglia was partially suppressed by the co-administration of rrIL-1ra (Fig. 6d). These data indicate that the co-administration of rrIL-1ra attenuated neuroinflammation and neuronal cell death in the hippocampus. Taken together, these results suggest that NG2 glial ablation-induced hippocampal neuronal apoptosis may be the result of the IL-1β pro-inflammatory pathway in activated microglia.

### NG2 glia-derived hepatocyte growth factor is required for the survival of hippocampal neurons

**Figure 7:**
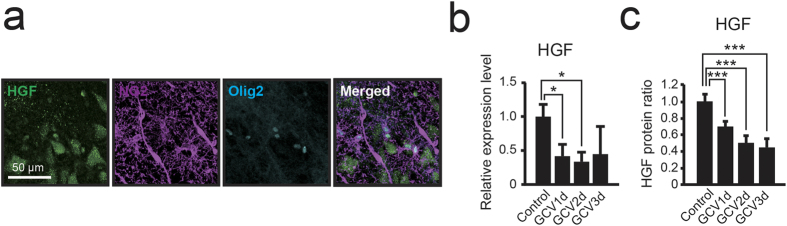
Ablation of NG2 glial cells induces the reduction of HGF in the hippocampus. (**a**) Confocal images of immunoreactivity for hepatocyte growth factor (HGF) (green), NG2 (magenta), and Olig2 (cyan) in NG2-HSVtk transgenic rats. (**b**) Relative expression levels of HGF mRNA in the hippocampus of NG2-HSVtk transgenic rats treated with vehicle (Control, the value as 1.0) or GCV for 1, 2, 3 days (GCV1d, GCV2d, GCV3d). (**c**) Relative protein abundance of HGF evaluated based on a densitometry analysis using ImageJ in the hippocampus of NG2-HSVtk transgenic rats treated with vehicle (Control, the value as 1.0) or GCV for 1, 2, or 3 days. The amount of total protein was used as a loading control. Mean ± SD, n = 3 animals (Control, GCV3d) or 4 animals (GCV1d, GCV2d); *p < 0.05 or ***p < 0.005, based on a one-way ANOVA followed by Tukey-Kramer test (**b**,**c**). Scale bar represents 50 μm (**a**).

To investigate the mechanism underlying NG2 glial cell regulation of neuronal survival and the IL-1β pro-inflammatory pathway in activated microglia, we investigated the expression of the multifunctional cytokine hepatocyte growth factor (HGF). HGF is expressed in cultured NG2 glial cells^35^,^36^, and such expression inhibits the inflammatory response via the suppression of IL-1β and IL-6 production^37^,^38^. In the present study, triple-immunohistochemical staining for HGF, NG2, and Olig2 revealed the expression of HGF in both NG2 glial cells and hippocampal neurons (Fig. 7a). Moreover, histological analysis revealed that microglia (CD11b expressing cells) and astrocytes (S100β expressing cells) in the hippocampus did not express HGF (Supplementary Fig. S5). These results suggest that the HGF expression originated in both NG2 glia and neurons in the hippocampus. Next, we investigated HGF mRNA and protein levels in both the intact and NG2 glia-ablated hippocampus. HGF mRNA expression was significantly decreased in the hippocampus beginning 1 day after the start of GCV infusion (Fig. 7b; p = 0.036). Western blot analysis indicated that HGF began to decrease significantly 1 day after the start of GCV infusion (Fig. 7c; p = 0.0048). The alteration of HGF protein expression correlated with the mRNA expression level of HGF. Such results suggest that NG2 glial cells might be an important source of HGF in the hippocampus.

**Figure 8:**
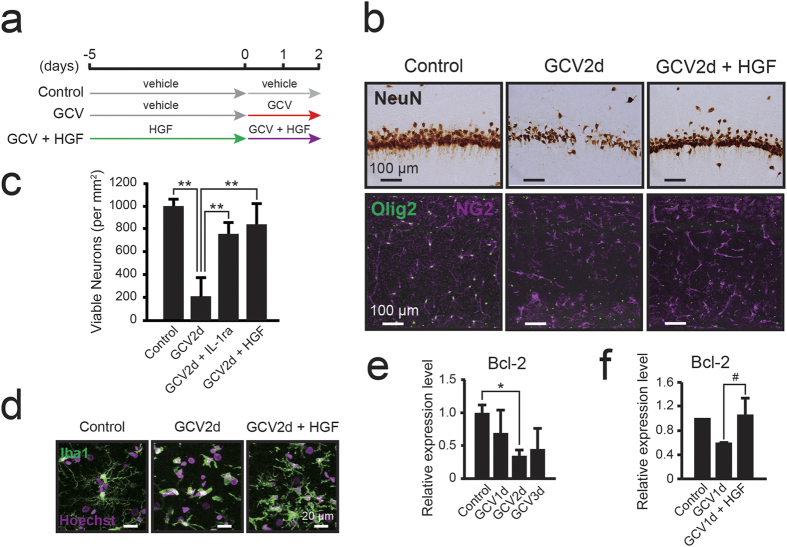
NG2 glial cell-derived HGF supports the survival of hippocampal neurons. (**a**) Timeline of the experimental design for the treatment with mouse recombinant HGF (mrHGF). (**b**) Bright-field immunohistochemical observations of NeuN in the hippocampal CA1 region of NG2-HSVtk transgenic rats treated with vehicle (Control), GCV for 2 days (GCV2d), or co-administration of GCV and HGF (4.3 μg/day) for 2 days (GCV2d + HGF). (**c**) The number of viable neurons in NG2-HSVtk transgenic rats treated with vehicle (n = 2), GCV for 2 days (n = 3), co-administration of GCV and IL-1ra (GCV2d + IL-1ra, n = 3), or co-administration of GCV and HGF (GCV2d + HGF, n = 4). (**d**) Confocal images showing microglia (Iba1, green) in the hippocampal CA1 region of NG2-HSVtk transgenic rats treated with vehicle, GCV for 2 days, or co-administration of GCV and HGF for 2 days. Hoechst, cell nuclear staining. (**e**) Relative expression levels of Bcl-2 mRNA in the hippocampus of NG2-HSVtk transgenic rats treated with vehicle (Control, value set as 1.0) or GCV for 1, 2, or 3 days. n = 3 animals (Control, GCV3d) and 5 animals (GCV1d, GCV2d). (**f**) Relative expression levels of Bcl-2 mRNA in the hippocampi of rats treated with vehicle (Control, value set as 1.0, n = 2), GCV for 1 days (n = 3), or co-administration of GCV and HGF for 1 day (n = 3). Mean ± SD; *p < 0.05 or **p < 0.01, based on a one-way ANOVA followed by Tukey-Kramer test (**c**,**e**). ^#^p = 0.05, based on a Student’s t-test analysis (**f**). Scale bars represent 100 μm (**b**) or 20 μm (**d**).

To determine whether the reduction in HGF due to the ablation of NG2 glial cells exacerbated the apoptotic cell death of hippocampal neurons, we co-administered GCV and mouse recombinant HGF (mrHGF), which shows a 99% homology with rat HGF, in the hippocampus following ablation of NG2 glial cells (Fig. 8a). Immunohistochemical staining for NeuN revealed significant preservation of hippocampal neurons 2 days after the start dual GCV/HGF treatment (viable neurons, 839.1 ± 177.0 cells/mm^2^; p = 0.002) when compared with that observed for GCV alone (viable neurons, 207.9 ± 165.9 cells/mm^2^) (Fig. 8b and c). Further, activation of microglia was strongly attenuated by the co-administration of mrHGF in the NG2 glia-ablated hippocampus (Fig. 8d).

HGF is a robust neurotrophic factor for various types of neurons^39^, and expression of the HGF receptor (c-Met) has been observed in hippocampal neurons *in vivo*^35^,^36^,^40^,^41^. Furthermore, fibroblast growth factor-2 (FGF-2) from glial cells protects hippocampal neurons from several types of stress via upregulation of the apoptosis-suppressor gene B-cell lymphoma 2 (Bcl-2)^42^. Bcl-2 has been identified as a critical regulator of hippocampal neuron viability^43^. Indeed, the expression of Bcl-2 gradually decreased after the start of GCV infusion (Fig. 8e; p = 0.026). Thus, we evaluated the influence of HGF administration on the expression of Bcl-2. Real time PCR analysis revealed that the expression of Bcl-2 decreased following NG2 glial cell ablation, while treatment with HGF returned the level of Bcl-2 expression to baseline (Fig. 8f; p = 0.05). These results suggest that NG2 glial cells seem to support the survival of hippocampal neurons through the upregulation of HGF-induced Bcl-2. Taken together, our findings suggest that NG2 glial cells have the potential to suppress neuroinflammation and support the survival of hippocampal neurons through the bifunctional activity of HGF.

## Discussion

Until recently, NG2 glial cells were believed to be responsible for only the production of mature oligodendrocytes in the CNS. However, recent studies have revealed that NG2 glial cells actively migrate and proliferate to restore their density in the adult CNS^2^. NG2 glial cells also receive synaptic input and modulate activity throughout the neuronal network^10^,^11^,^14^. In addition, selectively removing mutant genes that are responsible for amyotrophic lateral sclerosis (ALS) from NG2 glial cells delayed disease onset and progression in a mouse model of ALS^19^. Such findings support our hypothesis that NG2 glial cells are involved in the maintenance of neuronal function and survival of neurons in the adult CNS, as well as in the production of mature glial cells. To investigate these potential roles of NG2 glia, we developed transgenic rats expressing HSVtk in NG2 glial cells and selectively ablated the NG2 glial cells in the CNS (Figs 1 and 2). We subsequently observed that ablation of NG2 glial cells induced excessive neuroinflammation triggered by the activation of microglia, resulting in neuronal impairment and cell death, particularly in the hippocampus (Figs 3, 4, 5 and 6). Moreover, it is likely that HGF secreted from NG2 glia supports the survival of hippocampal neurons (Figs 7 and 8). HGF is one of the most potent survival-promoting factors for neurons, suppressing inflammation and activating the anti-apoptotic pathway (Supplementary Fig. S6). Further studies have indicated that HGF exerts anti-inflammatory effects by increasing the expression of IL-1ra in hepatocytes^44^. In the present study, we also observed that IL-1ra attenuated NG2 glial cell ablation-induced neuronal cell death (Fig. 6). It is possible that HGF suppresses neuroinflammation via the production of IL-1ra in the CNS. Thus, NG2 glia-derived HGF may be a critical factor in the development of new therapies for neurodegenerative diseases. However, the mechanism of HGF production in NG2 glia in response to inflammation and physiological function of NG2 glia-derived HGF remains unclear. Further research is required to elucidate the precise molecular mechanism.

A recent study investigated the ablation of NG2 glial cells in NG2Cre/inducible diphtheria toxin receptor (iDTR) transgenic mice^15^. However, only partial ablation of NG2 glial cells was observed in the cortex, along with the repopulation activity of the cells. Studies involving NG2 glial cell ablation in NG2Cre/iDTR transgenic mice also likely ablate pericytes and mature oligodendrocytes due to the degeneration of all iDTR-expressing cells, including the non-proliferative cells. In the present study, we used an HSVtk/GCV system to selectively ablate NG2 glial cells. HSVtk converts GCV into a toxic triphosphate molecule that can be incorporated into the genome and terminate DNA synthesis. Thus, this system is preferable due to the ability of GCV administration to ablate proliferative HSVtk positive cells.

In general, the proliferating rate of NG2 glial cells is less than 1.0% in adult rats^16^. However, NG2 glial cells were completely ablated within a few days in surrounding regions of the lateral ventricle/hippocampus in NG2-HSVtk transgenic rats. (Figure 2b and c), suggesting that almost all NG2 glial cells initiated proliferative activity within a few days. To address this issue, we assessed proliferative activity after the start of GCV infusion via BrdU administration, which is commonly used as a marker of proliferating cells. The results of this experiment indicate that GCV treatment dramatically increases the number of BrdU-incorporated cells in the hippocampus compared with the control treatment. Moreover, NG2 and BrdU immunostaining showed that 88.8% of BrdU-labeled cells were NG2 positive cells [95/107 cells] (Supplementary Fig. S7). As aforementioned, NG2 glial cells actively proliferate to restore their density in response to focal reductions in cell number^5^,^15^. The ablation of NG2 glial cells following GCV administration could have activated proliferative activity in the surrounding NG2 glial cells.

NG2 glial cells complete the differentiation into mature oligodendrocytes taking a few weeks^2^,^26^. Thus, we ablated NG2 glial cells within a few days using a conventional dose of GCV to eliminate the influences of dysfunctional differentiation. Such a rapid ablation of NG2 glial cells did not influence the number of oligodendrocytes in the hippocampus. Olig2+/NG2- cells are considered to be neural stem cells, immature oligodendrocytes, and mature oligodendrocytes, and neural stem cells are located in restricted regions, such as subventricular zone (SVZ) and subgranular zone (SGZ)^2^. To assess the effect of pharmacological ablation in oligodendrocyte lineage cells, we excluded the DG region and counted Olig2+/NG2- cells in the CA1, CA2, and CA3 regions of the hippocampus in NG2-HSVtk transgenic rats. Oligodendrocyte lineage cells were not significantly impaired at 1 day after the start of GCV infusion (148.1 ± 30.3 cells/mm^2^; p = 0.694) compared with the controls (159.3 ± 48.0 cells/mm^2^). This finding was likely because NG2 expression is lost during the differentiation of NG2 glial cells into mature oligodendrocytes. In the present study, mature oligodendrocytes did not express HSVtk (Fig. 1e).

Further, no obvious degeneration of pericytes was observed (Fig. 2d and e), likely due to the low proliferative activity of pericytes. A recent study has demonstrated that age-related loss of pericytes results in a progressive vascular-mediated neurodegeneration following the breakdown of the blood-brain barrier (BBB)^27^. In the present study, we assessed BBB permeability using Evans Blue. No extravasation was observed within 3 days after GCV treatment (Supplementary Fig. S8a). Blood-derived thrombin is a mediator of neurotoxicity and diffuses into the brain parenchyma following BBB breakdown^27^. Therefore, we assessed the leakage of thrombin using an immunoblot analysis in the hippocampus and observed little or no thrombin extravasation (Supplementary Fig. S8b and c; p = 0.61). Moreover, no infiltration of hematopoietic cells, such myeloid-derived suppressor cells^45^,^46^ was observed within 3 days after GCV treatment (Supplementary Fig. S9). Such observations in the present study suggest limited involvement of pericytes and the BBB in neuronal cell death.

In our study, hippocampal neurons underwent cell death via the ablation of NG2 glial cells. Use of the HSVtk/GCV system may have impaired neighboring cells due to the production of cytotoxic GCV by targeted cells, and subsequent cytotoxic GCV transmission through gap junctions, which is a phenomenon known as the bystander effect^47^. However, this phenomenon is thought to have little affect on non-proliferative cells such as hippocampal neurons. Further, administration of IL-1ra or HGF preserved neurons even in the NG2 glia-ablated hippocampus (Figs 6b and 8b,c). Such data in the present study suggest that the bystander effect did not induce neuronal impairment.

In addition, conventional cell ablation methods, such as the HSVtk/GCV and DTR/DT systems require intracerebroventricular infusion of pharmacological agents, a procedure that results in a small wound that may trigger neuroinflammation (Supplementary Fig. S10). Further, the damage-associated molecular patterns (DAMPs) from GCV-treated cells have been suggested to produce such neuroinflammation^48^. However, such a slight neuroinflammatory response likely does not induce acute neuronal degeneration. Although selective ablation of GFAP positive astrocytes led to rapid neuronal dysfunction, neuronal loss was observed even if microglia were eliminated in the adult CNS^49^. This result was observed because, according to Schereiner *et al*., astrocytes do not regulate the immune response, but maintain neuronal health through the regulation of redox homeostasis in the adult CNS^49^. Such data indicate that NG2 glia and astrocytes possess different neuroprotective abilities, and that cell ablation can reveal cell-specific function without a non-specific effect of the ablation of targeted cells.

Treatment with a low amount of GCV could ablate almost all of the NG2 glial cells for 1 week (Fig. 2b and c). Such gradual ablation of NG2 glial cells also led to neurodegeneration in the hippocampus depending on the abundance of NG2 glia (Fig. 3b,c). These results suggested that NG2 glial cell ablation-induced neuronal degeneration is comparable with the effect of astrocyte ablation on neurons^49^. Moreover, we hardly observed the degeneration of hippocampal neurons at 2 days after the start of GCV infusion at doses of ~1.0 mg/ml (Fig. 3b,c). However, low GCV treatment for 2 days initiated the activation of microglia (Supplementary Fig. S11). To clarify the mechanism of neurodegeneration, we suppressed the activation of microglia via co-treatment with minocycline. The treatment with minocycline partially suppressed the NG2 glial cell ablation-induced neurodegeneration (Fig. 5b,c). Such results suggest that the ablation of NG2 glia triggered the inflammatory response in microglia, resulting in neuronal degeneration and loss.

Our results support the idea that HGF is an important factor for the survival of hippocampal neurons and the suppression of neuroinflammation. However, additional factors produced by NG2 glial cells may be involved in these processes. Indeed, the loss of NG2 glia-derived FGF-2 led to the dysfunction of neurons in the prefrontal cortex^50^. In our study, microarray analysis revealed that the expression of FGF-2 was not decreased in the hippocampus following the ablation of NG2 glia (Supplementary Table S1). Such data suggest that NG2 glia might possess a different function in the hippocampus compared to the prefrontal cortex. In addition, we observed decreases in the anti-inflammatory cytokine IL-10, insulin-like growth factor 2 (IGF2), and fibroblast growth factor (FGF13) in the hippocampus following the ablation of NG2 glial cells (Supplementary Table S1). Among these cytokines and growth factors, IL-10 may play an important role in the regulation of neuroinflammation. Previous research reports that IL-10 enhances the M2 phenotype of macrophages, encouraging tissue repair through activation of the IL-4 pathway^51^. In the present study, IL-1ra also supported the survival of neurons and suppressed neuroinflammation (Fig. 6). However, the mechanism underlying the regulation of IL-1ra expression in the CNS has not been elucidated. A recent study found that HGF exerts anti-inflammatory effects via signal transduction modulation leading to the increased expression of IL-1ra^44^. Such a result suggests that NG2 glia-derived HGF may regulate the expression of IL-1ra. Similarly, hematopoietic progenitor cells regulate the inflammatory response through versatile cytokines^52^. Taken together, our results and previous studies suggest that progenitor cells such as NG2 glial cells may play a role in the general regulation of the inflammatory response.

Neurons are vulnerable to the neuroinflammation that is associated with several neurodegenerative diseases^53^ such as multiple sclerosis^54^, Alzheimer’s disease, Parkinson’s disease, and ALS. In addition, neuroinflammation has been associated with various mental disorders, including schizophrenia^55^ and chronic fatigue syndrome^56^. Therefore, understanding the mechanism underlying the role of NG2 glial cells in the regulation of the immune system in the CNS may highlight the biological importance of maintaining the CNS and lead to the development of new therapeutic approaches for the chronic neuroinflammation that is observed in neurodegenerative diseases, demyelinating diseases, and mental disorders, and the functional deterioration of the CNS due to aging.

## Methods

### Generation of NG2-HSVtk transgenic rats

We used bacterial artificial chromosome (BAC) modification to generate transgenic rats that express HSVtk specifically in NG2-expressing cells. We obtained a 205.3 kb BAC clone (clone no. CH230–428C7) in pTARBAC2.1 vector from the Children’s Hospital Oakland Research Institute. This BAC vector contained the entire 45.6 kb Cspg4 (Ng2) gene that was flanked by 76.2 kb and 94.1 kb of additional sequences on the 5′ and 3′ ends of the gene, respectively. To prepare the NG2-HSVtk transgene, HSVtk cDNA was obtained from Dr. Tsuyoshi Tahara. The HSVtk cDNA was correctly inserted between the first and last exon of the NG2 gene in the BAC clone. The linearized NG2-HSVtk transgene was microinjected into fertilized oocytes of Wister rats. The NG2 transgenic rats were generated at PhoenixBio Co., Ltd. (Tochigi, Japan). Founder rats were identified based on PCR using forward (5′-GTAATGACAAGCGCCCAGTAT-3′) and reverse (5′-ATGCTGCCCATAAGGTATCG-3′) primers from the HSVtk cDNA. All transgenic rats, as well as control animals, were obtained by mating heterozygous females with non-transgenic males of the respective lines. Thus, transgenic and non-transgenic control animals were derived from the same inbred line and had similar genetic backgrounds. The genotype was confirmed postmortem using immunohistochemical detection of HSVtk.

### Animals

We used adult male NG2-HSVtk transgenic rats (12 to 35 weeks old) and adult male Wistar rats (SLC, Hamamatsu, Japan; 15 weeks old). All experimental protocols were approved by the Ethics Committee on Animal Care and Use of the RIKEN Center for Life Science Technologies (MAH21-17-15), and were performed in accordance with the Principles of Laboratory Animal Care. Rats were housed at a constant temperature (22–23 °C) and humidity (50–60%) and maintained under a 12-hour light/dark cycle with free access to food and water.

### Surgical procedure for intracerebroventricular infusion using a microinfusion pump

For implantation of the pump and cannula, adult rats were anesthetized with isoflurane (Pfizer, Groton, CT, USA) and fixed to a stereotaxic frame after a loss of the paw withdrawal reflex. A chronic intracerebroventricular (i.c.v.) cannula (Brain Infusion Kit 1; Alzet, Cupertino, CA) was inserted into the left lateral ventricle of the brain under isoflurane anesthesia. The cannula was stereotaxically positioned in the lateral ventricle according to the Paxinos and Watson rat brain atlas at the following coordinates from bregma: anterior-posterior (AP), −0.8 mm; medial-lateral (ML), 1.5 mm; and dorsal ventral (DV), −3.2 mm. The cannula was secured to the skull of the animal with stainless steel screws and dental cement. The main body of the “iPRECIO” microinfusion pump (Model: SMP-200, Primetech Corporation, Tokyo, Japan) was implanted subcutaneously under the back of the neck, and the iPRECIO catheter was connected to the cannula. After closing the incision, penicillin G potassium (6000 U/day, Meiji Seika Pharma Co., Ltd., Tokyo, Japan) was injected intramuscularly for infection control. Sham-operated rats received a similar surgical operation without the implantation of iPRECIO.

Following surgery, all rats were individually housed for the remainder of the experiment. The infusion start time, infusion end time, and infusion rate were programmed with the iPRECIO Management Software Ver. 1.3 (Primetech Corporation, Tokyo, Japan). The infusion rate was set at 1.0 μl/h for all i.c.v. infusion procedures. The iPRECIO was filled with either vehicle or ganciclovir solution (Mitsubishi Tanabe Pharma Corporation, Osaka, Japan) at concentrations of 0.01, 0.1, 0.5, 1.0, and 10 mg/ml. A GCV dose of 10 mg/ml (10 mg/ml × 24 μl/day ÷ 4.5 ≈ 53.3 μg/day) in transgenic rats is equivalent to the GCV treatments in mice discussed in the aforementioned reports (about 30~300 μg/day)^22^,^23^,^24^. Solutions were continuously infused into the lateral ventricle for 1, 2, 3, or 7 days. Ablation of NG2-expressing cells was confirmed using immunohistochemistry. Wild-type littermates were also infused with ganciclovir to serve as control animals. No specific adverse side effects of ganciclovir were observed.

The i.c.v. infusion of IL-1ra, HGF, or rrIL-1ra (R&D Systems, Inc.; dissolved in 10-mM phosphate-buffered saline [PBS; pH 7.4, Nakarai Tesque, Kyoto, Japan] containing 0.1% bovine serum albumin [BSA]) at a dose of 1 μg/day, and mrHGF (R&D Systems, Inc.; dissolved in 10-mM PBS containing 0.1% BSA) at a dose of 4.3 μg/day or vehicle, was maintained for 5 days following surgery. Subsequently, the solution in the reservoir of the iPRECIO was changed to include each substance at the same concentration or vehicle in addition to ganciclovir at a concentration of 10 mg/ml. The infusion rate remained unchanged. Procedures for changing substances in the reservoir of iPRECIO were conducted under light anesthesia using isoflurane inhalation applied through a facemask.

### 5′-Bromodeoxyuridine administration

For labeling of proliferating cells, adult rats were intraperitoneally injected with 5′-bromo-deoxyuridine (BrdU; 50 mg/kg, Sigma-Aldrich, St Louis, MO, USA). BrdU was injected twice a day for 1, 2, or 3 days after the start of ganciclovir infusion.

### Minocycline administration

Adult rats were intraperitoneally injected with minocycline at a dose of 45 mg/kg/day (Sigma-Aldrich, St Louis, MO, USA). Minocycline was injected once a day for 2 days after the start of ganciclovir infusion.

### Immunohistochemical staining

Rats were deeply anesthetized with pentobarbital and perfused transcardially with 4% formaldehyde (Millipore, Billerica, MA, USA) in 10-mM PBS 1, 2, 3, or 7 days after the onset of infusion with either vehicle or ganciclovir. Brains were removed, postfixed overnight in 4% formaldehyde buffered with 10-mM PBS at 4 °C, and then immersed in 15% and 30% (w⁄v) sucrose solution. Coronal brain sections (30-μm thickness) were prepared using a cryostat (Microm HM 560, Microedge Instrument Inc.). For BrdU staining, the brain sections were pre-incubated in 2 N HCl at 37 °C for 30 minutes and rinsed in 0.1-M boric acid (pH 8.5) at 25 °C for 10 minutes. For multiplex-immunostaining, unmasking for PDGFRβ and Glut1 was performed using boiling for 10 min in 1-mM EDTA buffer (pH 8.0, Nakarai Tesque, Kyoto, Japan) in a pressure cooker (T-fal CLipso, Groupe SEB). Coronal sections were blocked with blocking buffer (5% donkey serum and 0.3% Triton X-100 in PBS) and subsequently incubated with several primary antibodies at 4 °C for 12–36 h. The primary antibodies used in this study were as follows: monoclonal mouse anti-neuronal nuclei (NeuN) IgG (1:200, Millipore); monoclonal mouse anti-NG2 IgG (1:200, Millipore); polyclonal rabbit anti-NG2 IgG (1:200, Millipore); and polyclonal rabbit anti-GFAP IgG (1:100, Sigma); polyclonal rabbit anti-Iba1 IgG (1:200, Wako, Cape Charles, VA, USA); polyclonal rabbit anti- PDGFRβ IgG (1:100, Santa Cruz, Dallas, TX, USA); polyclonal goat anti-Glut1 IgG (1:100, Santa Cruz); polyclonal goat anti-HSVtk IgG (1:100, Santa Cruz); polyclonal rabbit anti-Olig2 IgG (1:500, Millipore); polyclonal rabbit anti-TNFR1 IgG (1:100, Santa Cruz), rabbit polyclonal anti-HGFα IgG (1:100, Santa Cruz); monoclonal mouse anti-MBP IgG (1:200, BioLegend, San Diego, CA, USA); rabbit polyclonal anti-MAP2 IgG (1:200, Millipore); monoclonal rat anti-BrdU IgG (1:500, AbD Serotec); monoclonal mouse anti-S100β IgG (1:500, Sigma); monoclonal rat anti-Ly6G IgG (1:500, Abcam). After washing for 30 min (three washes of 10 min each) with 0.3% Triton X-100 in PBS, brain sections were incubated in the appropriate secondary antibodies conjugated with either Cy2, Cy3, or Cy5 (1:200, Jackson ImmunoResearch, West Grove, PA, USA) at room temperature for 1 h and washed with PBS with Tween 20 (PBST) for 30 min. Some of the stained sections were mounted with solution containing Hoechst (1:1000, Dojindo Laboratories, Tokyo, Japan) and then examined using the confocal laser microscope (Digital Eclipse C1; Nikon, Tokyo, Japan). Apoptosis in the tissue was evaluated using the Apoptag Kit (Millipore), which detects nick-ended DNA fragments present in apoptotic cells.

### Real time PCR

Rats were deeply anesthetized with pentobarbital and perfused transcardially with 10-mM phosphate-buffered saline (PBS; pH 7.4) 1, 2, or 3 days after the onset of infusion with either vehicle or ganciclovir. After the perfusion, brains were quickly removed and placed on ice. The parietal cortex, striatum, hippocampus, hypothalamus, and amygdala on the ipsilateral side of the i.c.v. infusion were dissected, and the samples were incubated overnight in RNAlater (Thermo Fisher Scientific, Waltham, MA, USA), frozen with a dry ice, and stored at −80 °C. Total RNA was isolated from brain tissue using the ISOGEN Kit (Nippon Gene, Tokyo, Japan) according to the manufacturer’s protocol and resuspended in RNase-free water. The concentration of the total RNA was measured by calculating absorbance at a wavelength of 260 nm. The cDNA was synthesized from 1 μg of total RNA using reverse transcriptase with gDNA eraser (Takara Bio, Shiga, Japan). Complementary DNA (20 ng) was amplified using real time PCR. Real time PCR was performed in the Thermal Cycler Dice Real Time System (Takara Bio), using the KAPA SYBR qPCR Master Mix (Kapa Biosystems, Boston, MA). The PCR reaction began with denaturation at 95 °C for 3 min, followed by 45 cycles at 95 °C for 3 sec, 60 °C for 20 sec, and a final extension at 60 °C for 10 min. The relative expression of IL-1β, IL-6, TNFα, Bcl-2, and HGF mRNA were calculated after normalization to Rsp18 mRNA as an internal control. The primer sequences for amplification of cDNA were as follows: rat IL-1β, (sense) 5′-CCCTGAACTCAACTGTGAAATAGCA-3′ and (antisense) 5′-CCCAAGTCAAGGGCTTGGAA-3′; rat IL-6, (sense) 5′-ATTGTATGAACAGCGATGATGCAC-3′ and (antisense) 5′-CCAGGTAGAAACGGAACTCCAGA-3′; rat TNFα, (sense) 5′-TCAGTTCCATGGCCCAGAC-3′ and (antisense) 5′-GTTGTCTTTGAGATCCATGCCATT-3′; rat Bcl-2, (sense) 5′-TTGAGTTCGGTGGGGTCATG-3′ and (antisense) 5′-TCAGTCATCCACAGAGCGATG-3′; rat Rps18, (sense) 5′-CTTCCACAGGAGGCCTACAC-3′ and (antisense) 5′-GATGGTGATCACACGCTCCA-3′; and rat HGF, (Nihon Gene Research Laboratories Inc., Miyagi, Japan).

### Microarray analysis

Total RNA was isolated from the hippocampi of rats infused with vehicle or ganciclovir for 1 day. The integrity of the RNA was assessed using electrophoresis (Agilent 2100 Bioanalyzer, Santa Clara, CA). Expression profiling was performed by Takara Bio Inc. using SurePrint G3 Rat GE 8 × 60 K Microarray (Agilent Technologies).

### NG2 glia (Olig2, NG2, and HSV-TK positive) counting

For quantification of the number of NG2 glial cells, threshold processing was performed separately for Olig2, NG2, and HSV-TK signals. The overlapping signals in all three immunoreactivities were analyzed and counted using the NIH ImageJ Area measurement and the Cell Counter plug-in analysis tool to obtain the number of NG2 glial cells. At least four fields from the CA1, CA2, CA3, and DG regions of the hippocampus were analyzed in three randomly selected nonadjacent sections (~100 μm apart) from each animal.

### Glut1-positive pericyte coverage

For evaluation of the pericyte coverage of blood vessels, immunoreactive signals for Glut1 from endothelial cells and those for PDGFRb from pericytes were separately subjected to threshold processing. Areas occupied by the respective signals were analyzed using the NIH Image J Area measurement tool. Quantification of the pericyte coverage around the blood vessels was determined by obtaining the percentage (%) of the PDGFRb-immunopositive pericyte surface area covering the Glut1-positive capillary surface area in each defined field (650 × 650 μm)^27^. Four fields from the CA1, CA2, CA3, and DG regions of the hippocampus were analyzed in three randomly selected nonadjacent sections (~100 μm apart) from each animal.

### NeuN-positive neuronal nuclei counting

NeuN-positive neurons were quantified using the Image J Cell Counter analysis tool. At least four fields from the CA1, CA2, CA3, and DG regions of the hippocampus were analyzed in three randomly selected nonadjacent sections (~100 μm apart) from each animal.

### Evans blue extravasation

Rats were anesthetized with 4% isoflurane and injected intravenously with 4% Evans blue in saline (1 ml/kg, Tokyo Chemical Industry Co., Ltd., Tokyo, Japan). Rats were perfused with PBS through the left heart ventricle 1 hour after Evans blue administration. Brains were removed and photographed with a digital camera (PowerShot SX280 HS; Canon Inc., Tokyo, Japan).

### Western blot analysis

Total protein was isolated from hippocampal tissue using the ISOGEN kit and was lysed in ice-cold lysis buffer comprised of 50 mM Tris-HCl (pH 7.4), 150 mM NaCl, 1 mM EDTA, 1% Triton X-100, 0.25% Sodium deoxycholate, 0.1% SDS, and cOmplete protease inhibitor cocktail (Roche, Indianapolis, IA, USA). Equal protein amounts from total cell extracts were mixed with a sample buffer containing mercaptoethanol and heated at 97 °C for 10 minutes. Protein samples were separated using SDS-polyacrylamide gel electrophoresis and blotted onto Immobilon-P transfer membranes (Millipore). Membranes were blocked with 5% skim milk in PBS containing 0.05% Tween-20 and incubated with polyclonal rabbit anti-NG2 IgG (1:200, Chemicon, Billerica MA, USA); polyclonal goat anti-Thrombin HC IgG (1:50, Santa Cruz); rabbit polyclonal anti-HGFα IgG (1:100, Santa Cruz); mouse monoclonal horseradish peroxidase (HRP)-conjugated anti-α-tubulin IgG (1:1000, Proteintech); and goat polyclonal HRP-conjugated anti-GAPDH IgG (1:1000, Santa Cruz) overnight at 4 °C. After washing, membranes were incubated with HRP-linked antibody to mouse, rabbit (1:2000, GE Healthcare Life Science, Pittsburgh, PA, USA), or goat IgG (1:2000, Santa Cruz). Detection was performed with an ECL detection regent (GE Healthcare) using LAS-3000 (Fujifilm, Tokyo, Japan), and the intensity of each protein band was quantitated using ImageJ.

## Additional Information

**How to cite this article**: Nakano, M. *et al*. NG2 glial cells regulate neuroimmunological responses to maintain neuronal function and survival. *Sci. Rep.* **7**, 42041; doi: 10.1038/srep42041 (2017).

**Publisher's note:** Springer Nature remains neutral with regard to jurisdictional claims in published maps and institutional affiliations.

## Supplementary Material

Supplementary Figure
