# Supplementary material for: NG2 glial cells regulate neuroimmunological responses to maintain neuronal function and survival
Source: Sci Rep. 2017 Feb 14;7:42041. doi: 10.1038/srep42041 (PMC5307324; doi:10.1038/srep42041)
Supplement: Supplementary Figure [file srep42041-s1.pdf]

Supplementary Information

NG2 glial cells regulate neuroimmunological responses to maintain neuronal function and survival

Masayuki Nakano, Yasuhisa Tamura, Masanori Yamato, Satoshi Kume, Asami Eguchi, Kumi Takata, Yasuyoshi Watanabe, and Yosky Kataoka*

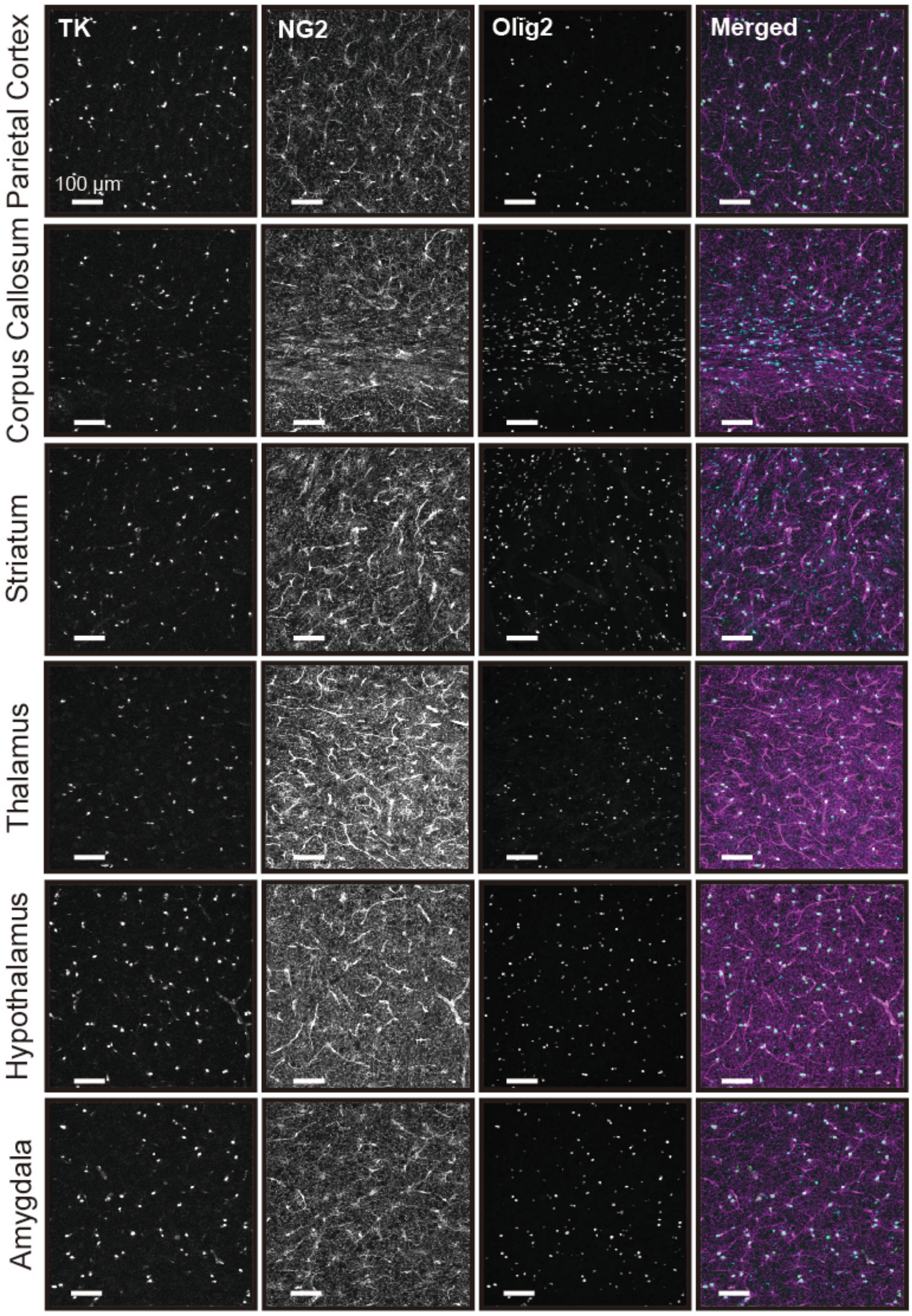

Supplementary Figure S1. HSVtk-expressing cells are widely distributed throughout the gray and white matter of the brain in NG2-HSVtk transgenic rats. Confocal images showing HSVtk (TK, green), NG2 (NG2, magenta), Olig2 (Olig2, cyan), and the merged condition (Merged, three colors) in the parietal cortex, corpus callosum, striatum, thalamus, hypothalamus, and amygdala of the brain in NG2-HSVtk transgenic rats. Scale bars represent 100 µm.

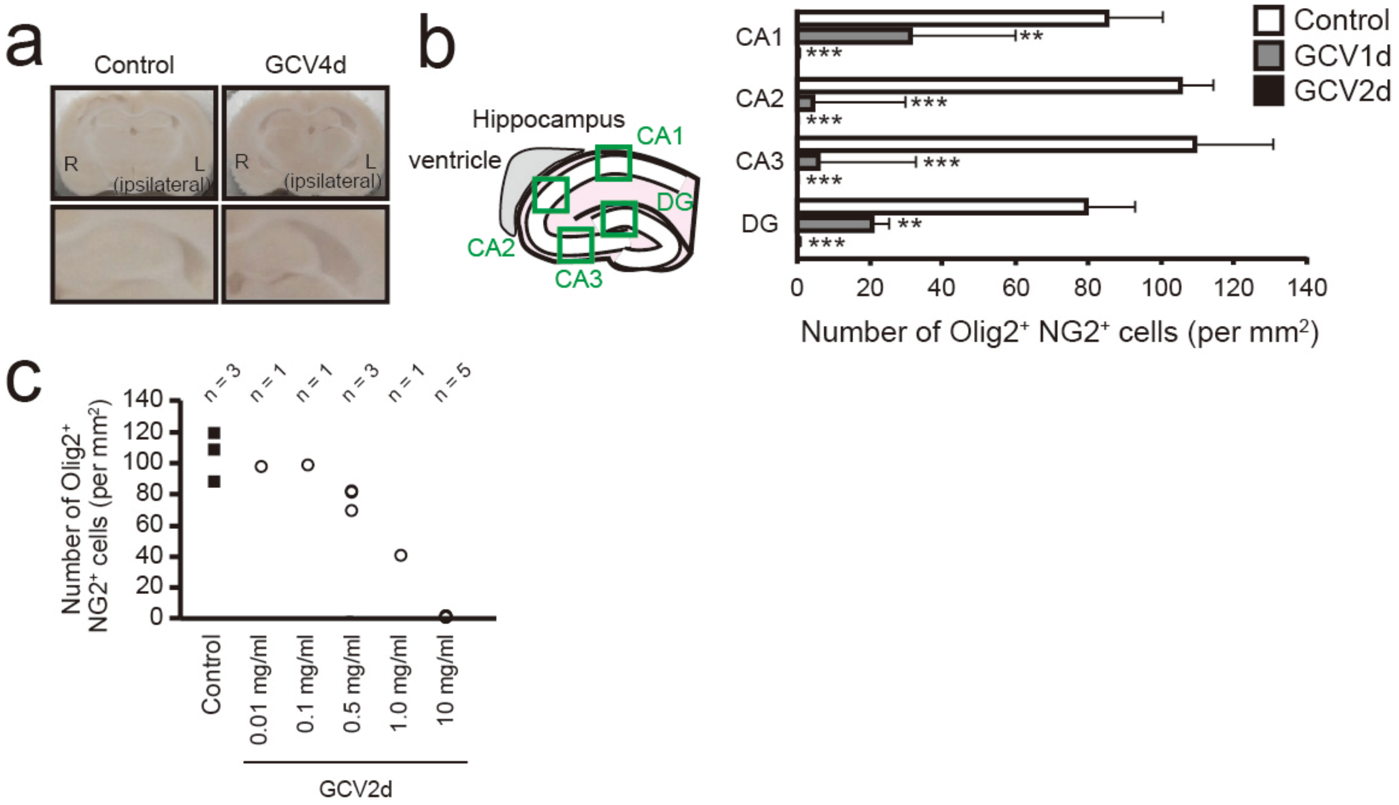

Supplementary Figure S2. Ablation of proliferating NG2 glial cells in each hippocampal region of NG2-HSVtk transgenic rats. (a) Coronal brain section from NG2-HSVtk transgenic rats treated with vehicle (Control, vehicle infusion) or GCV (GCV4d, 4 days after the start of the GCV infusion). 'R' represents right side and 'L' represents left side (ipsilateral side of intracerebroventricular infusion of vehicle or GCV). Lower panels are higher-magnified images in the ipsilateral hippocampus. (b) A schematic diagram of the CA1, CA2, CA3, and dentate gyrus (DG) regions of the hippocampus. The graph shows the number of NG2 glial cells in each hippocampal region of NG2-HSVtk transgenic rats after treatment with GCV at a dose of 10 mg/ml for 1 day (GCV1d), and 2 days (GCV2d), or with vehicle (Control). Mean ± SD, n = 3 rats (Control, GCV3d) and 5 rats (GCV1d, GCV2d); *p < 0.05, **p < 0.01 or ***p < 0.001, based on a one-way ANOVA followed by Tukey-Kramer test. (c) The number of NG2 glial cells (immunopositive cells for Olig2 and NG2) in animals treated with vehicle or GCV at doses of 0.01, 0.1, 0.5, 1.0, and 10 mg/ml for 2 days. Values for individual rats are shown with white circles (GCV) and black squares (Control); n = 1 rat [GCV (0.01 mg/ml) 2d, GCV (0.1 mg/ml) 2d, GCV (1.0 mg/ml) 2d], 3 rats [Control, GCV (0.5 mg/ml) 2d] or 5 rats [GCV (10 mg/ml) 2d].

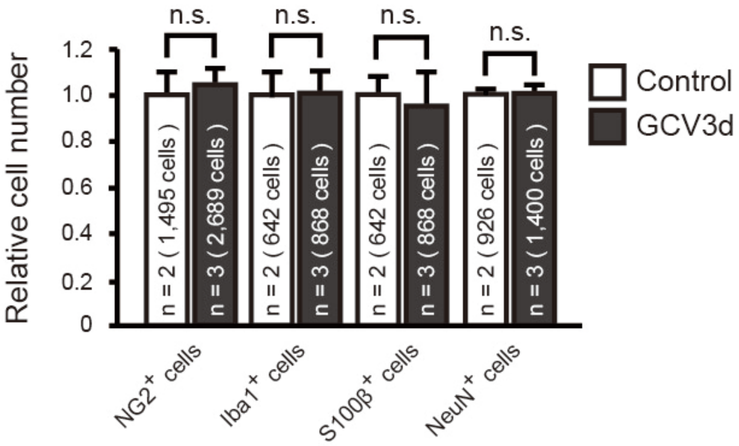

Supplementary Figure S3. The number of glial cells and neurons in wild-type (WT) rats treated with GCV. Relative cell number of NG2 glial cells and pericytes (immunopositive cells for NG2), microglia (immunopositive cells for Iba1), astrocytes (immunopositive cells for S100β), and neurons (immunopositive cells for NeuN) in the CA1 region of hippocampus in WT rats treated with vehicle (Control, value set as 1.0) or GCV at a dose of 10 mg/ml. The number of each cell in the bars indicates the total cell number. Mean ± SD, n = 2 rats (Control) or 3 rats (GCV3d); N.S., non-significant, p > 0.05, based on a Student' s t-test analysis.

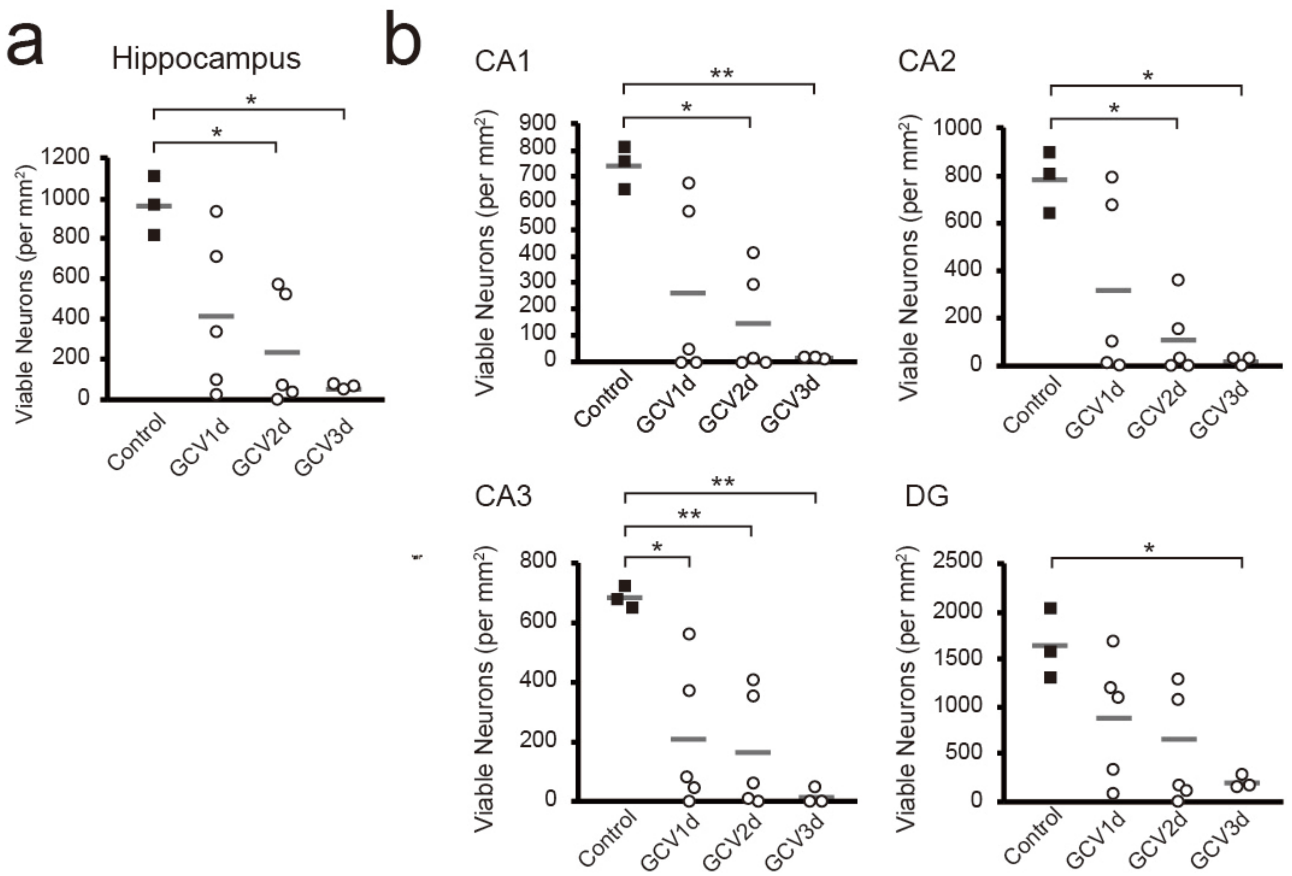

Supplementary Figure S4. Ablation of NG2 glial cells induces neuronal cell death in all regions of the hippocampus. The number of viable neurons in the CA1, CA2, CA3, and dentate gyrus (DG) regions of hippocampus in NG2-HSVtk transgenic rats treated with GCV for 1 (GCV1d), 2 days (GCV2d), and 3 days (GCV3d), or with vehicle for 3 days (Control). Values for individual rats are shown with white circles (GCV) and black squares (Control). Grey lines indicate the average values. Mean ± SD, n = 3 rats (Control, GCV3d) and 5 rats (GCV1d, GCV2d); *$p < 0.05$ or **$p < 0.01$, based on a one-way ANOVA followed by Tukey-Kramer test.

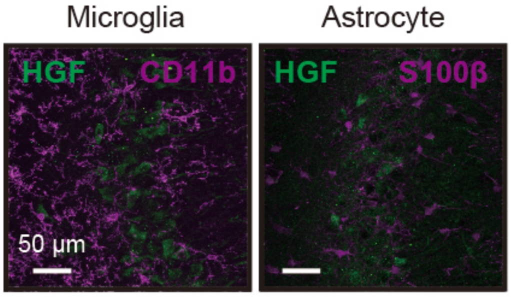

Supplementary Figure S5. HGF was not expressed in CD11b-immunopositive microglia or S100β-immunopositive astrocytes in the hippocampus. Immunohistochemical observations of HGF (green), CD11b (magenta in the left image), and S100β (magenta in the right image) in the hippocampal CA2 region of NG2-HSVtk transgenic rats treated with vehicle (Control). Scale bars represent 50 µm.

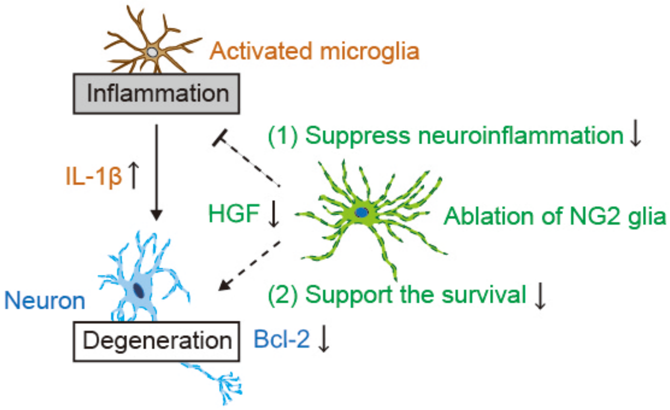

Supplementary Figure S6. The role of NG2 glial cells in the survival of hippocampal neurons. A conceptual diagram summarizes how NG2 glial cells suppress neuroinflammation by regulating the activation of microglia (1) and support the survival of hippocampal neurons by inducing the expression of Bcl-2 (2). Ablation of NG2 glia induces the loss of HGF, resulting in the neuroinflammation due to the activation of microglia and the degeneration of hippocampal neurons via down-regulating Bcl-2.

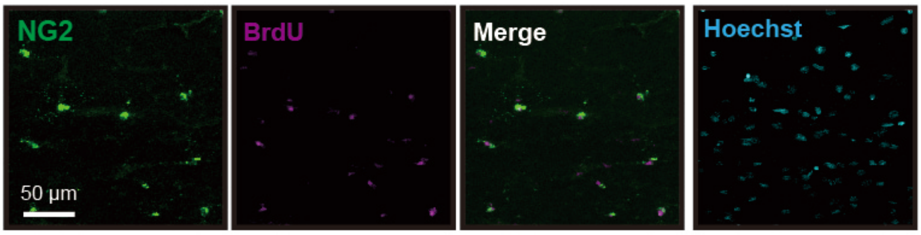

Supplementary Figure S7. BrdU-incorporation in NG2 glia-ablated hippocampus. To assess proliferative cells, rats were intraperitoneally injected with BrdU. BrdU was injected twice a day for 1 day after the start of GCV infusion in NG2-HSVtk transgenic rats. Confocal images showing NG2 (green), BrdU (magenta), and Hoechst (cyan) in the hippocampus of NG2-HSVtk transgenic rats that were treated with GCV for 1 day. Scale bar represents 50 µm.

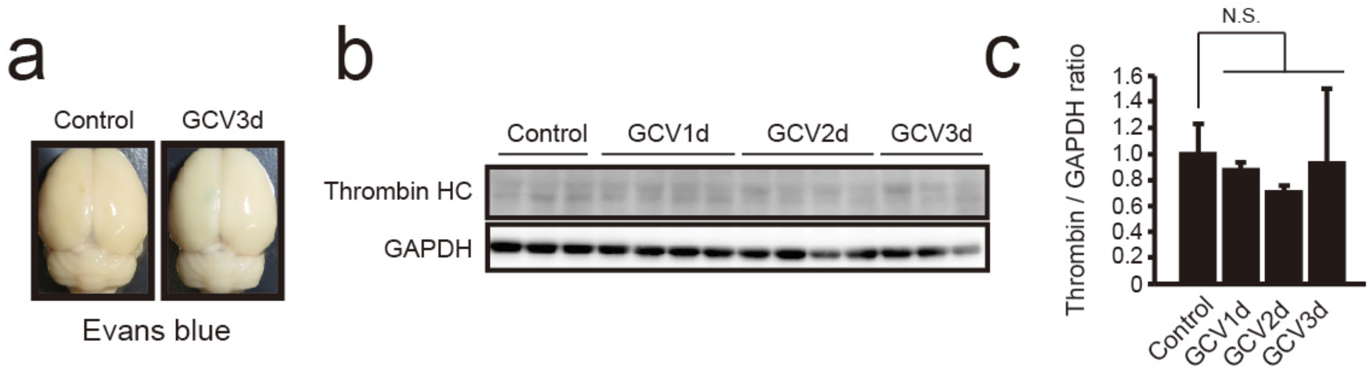

Supplementary Figure S8. The evaluation of blood-brain barrier leakage following NG2 glial cell ablation. (a) Photographs of whole brains taken from rats perfused with Evans blue (Control, vehicle infusion; GCV3d, GCV infusion for 3 days). (b) Immunoblotting of thrombin HC in the hippocampus of NG2-HSVtk transgenic rats treated with vehicle (Control, vehicle infusion) and GCV (GCV1d, GCV2d, or GCV3d; GCV infusion for 1, 2, or 3 days, respectively). GAPDH was used as a loading control. (c) Graphs show quantification of relative protein abundance of thrombin HC with densitometry analysis using ImageJ. Mean ± SD, n = 3 rats (Control, GCV3d) and 4 rats (GCV1d, GCV2d); N.S., non-significant, $p > 0.05$, based on a one-way ANOVA followed by Tukey-Kramer test.

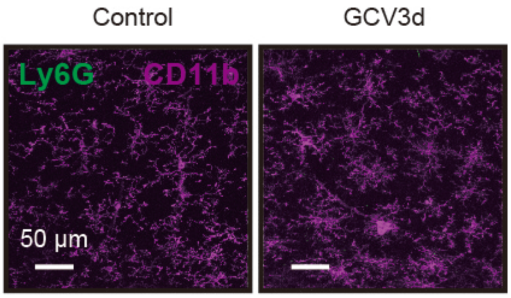

Supplementary Figure S9. The evaluation of hematopoietic-derived cell infiltration following NG2 glial cell ablation. Immunohistochemical observations of Ly6G (green) and CD11b (magenta) in the hippocampal CA1 region of NG2-HSVtk transgenic rats treated with vehicle (Control) and GCV for 3 days at a dose of 10 mg/ml (GCV3d). Scale bars represent 50 μm.

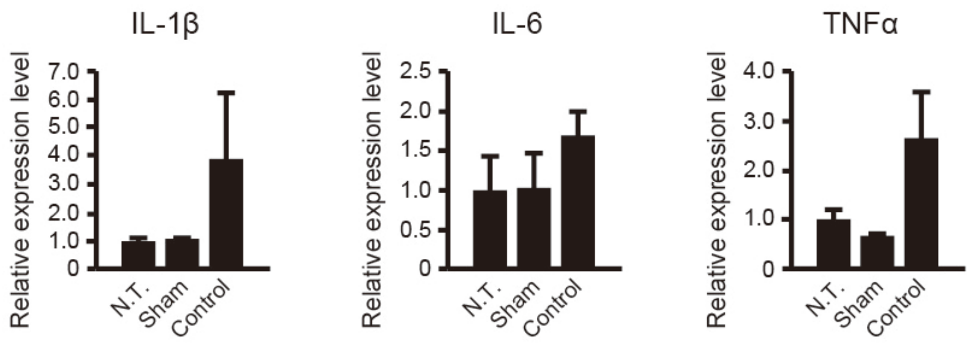

Supplementary Figure S10. Puncture wound induces a pro-inflammatory response in the hippocampus. Relative expressions of IL-1β, IL-6, and TNFα mRNAs in the hippocampus of NG2-HSVtk transgenic rats infused with vehicle (Control) compared with non-treatment rats (N.T., the value as 1.0), and sham operated rats (Sham). Sham-operated rats received similar surgical operation without the implantation of iPRECIO. Mean ± SD, n = 2 rats.

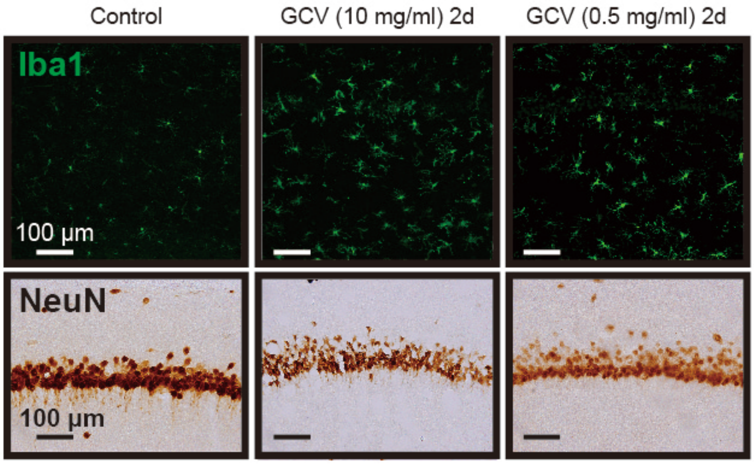

Supplementary Figure S11. Gradual ablation of NG2 glial cells initiated the activation of microglia without the neurodegeneration. Immunohistochemical observations of Iba1 (upper panels) and NeuN (lower panels) in the hippocampal CA1 region of NG2-HSVtk transgenic rats treated with vehicle (Control), or GCV for 2 days at a dose of 0.5 mg/ml [GCV (0.5 mg/ml) 2d] and 10 mg/ml [GCV (10 mg/ml) 2d]. Scale bars represent 100 µm.

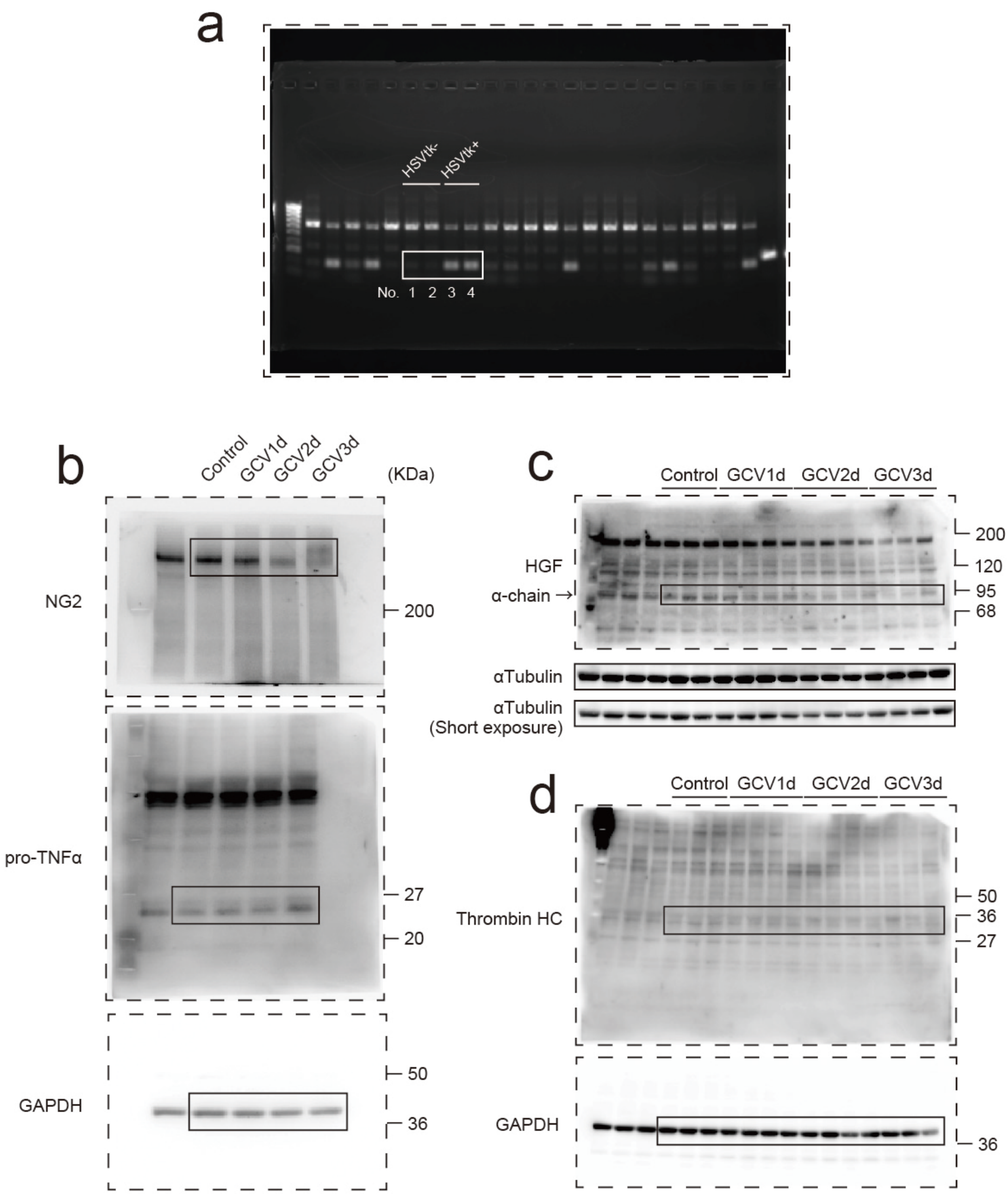

Supplementary Figure S12. Full - length gel electrophoresis and immunoblot images. (a) Uncropped image of gel electrophoresis in Figure 1b. (b) Uncropped images of blots in Figure 4c. (c) Uncropped images of blots in Figure 6c. (d) Uncropped images of blots in Supplementary Figure 5b. Cropping lines are indicated with squares. The dotted lines indicate the margins of the gels.

Gene Name	Gene expression (Log2 ratio) : Control vs GCV1d in Hippocampus
IL10	−2.05699513
IGF2	−1.646250666
HGF	−1.449844994
FGF13	−1.217689732
VCAM1	−0.981977485
VEGFc	−0.330858919
BDNF	−0.311610413
IL11	−0.302357769
EGF	−0.163742981
IGF1	−0.067282399
PDGFb	0.037528757
PDGFa	0.070925924
PDGFc	0.757163024
VEGFa	0.942310098
FGF2	0.951962424
TGFb1	2.119503418
ICAM1	2.879239995
CCL2	6.13988651

Supplementary Table S1. Alteration of gene expression levels of growth factors and cytokines in NG2 glia-ablated hippocampus. Gene expression levels of growth factors and cytokines in the hippocampus of NG2-HSVtk transgenic rats infused with GCV (GCV1d, GCV infusion for 1day) compared with vehicle (Control, vehicle infusion).

Primary antibodies for Immunohistochemical staining

Target Protein (or markers)	Host species	Provider	Catalog number	Dilution	Remarks
BrdU	rat	AbD serotec	OBT0030S	500	
GFAP	rabbit	Sigma	G9269	100	
Glut1	goat	Santa Cruz	sc-1605	100	
HGF	rabbit	Santa Cruz	sc-7949	100	
HGF	rabbit	Abcam	ab83760	100	
HSVtk	goat	Santa Cruz	sc-28037	100	1 mM EDTA buffer pH 8.0 in a pressure cooker
Iba1	rabbit	Wako	019-19741	200	1 mM EDTA buffer pH 8.0 in a pressure cooker
MAP2	rabbit	Millipore	AB5622	200	
MBP	mouse	Biolegend	94R-100	200	
NeuN	mouse	Millipore	MAB377	200	
NG2	mouse	Millipore	MAB5384	200	
NG2	rabbit	Millipore	AB5320	200	
Olig2	rabbit	Millipore	AB9610	500	
PDGFRβ	rabbit	Santa Cruz	sc-432	100	
TNFR1	rabbit	Santa Cruz	sc-8436	100	
S100β	mouse	Sigma	S2532	500	

Supplementary Table S2. Source and dilution of primary antibodies.

Target Gene	Forward	Reverse
(For qPCR)		
rat IL-1β	CCCTGAACTCAACTGTGAAATAGCA	CCCAAGTCAAGGGCTTGGAA
rat IL-6	ATTGTATGAACAGCGATGATGCAC	CCAGGTAGAAACGGAACTCCAGA
rat TNFα	TCAGTTCCATGGCCCAGAC	GTTGTCTTTGAGATCCATGCCATT
rat Bcl-2	TTGAGTTCGGTGGGGTCATG	TCAGTCATCCACAGAGCGATG
rat Rps18	CTTCCACAGGAGGCCTACAC	GATGGTGATCACACGCTCCA
(For Genotype)		
HSVtk	GTAATGACAAGCGCCCAGTAT	ATGCTGCCCATAAGGTATCG

Supplementary Table S3. List of primers used for qPCR and genotyping.
